# A toddler with systemic contact dermatitis caused by diabetes devices

**DOI:** 10.1002/ski2.234

**Published:** 2023-04-08

**Authors:** Anne Viktoria Lyngstadaas, Jan‐Øivind Holm, Lars Krogvold, Anne Karin Måløy, Christoffer Aam Ingvaldsen

**Affiliations:** ^1^ Department of Dermatology Rikshospitalet Oslo University Hospital Oslo Norway; ^2^ Institute of Clinical Medicine Faculty of Medicine University of Oslo Oslo Norway; ^3^ Division of Pediatric and Adolescent Medicine Oslo University Hospital Oslo Norway; ^4^ Department of Microbiology Akershus University Hospital Lørenskog Norway

## Abstract

Continuous glucose monitors (CGM) and insulin pumps have become the preferred treatment option for most young children and adolescents with type 1 diabetes (T1D), by avoiding fingerstick testing and providing real‐time glucose measurements. These medical devices and their adhesives contain substances which have been identified as being responsible for allergic contact dermatitis. We describe the case of a toddler who developed severe contact dermatitis from her diabetes devices, leading to secondary infections and hospital admissions. This was followed by the development of a symmetrical exanthema with retroauricular and glutaeal distribution. Patch tests were positive for isobornyl acrylate (IBOA) and 4‐tert‐butylcatechol (PTBC). Her symmetrical exanthema was interpreted as systemic contact dermatitis due to IBOA and PTBC in her diabetes devices. We suspect that systemic contact dermatitis is an underreported complication in diabetic patients.

## Graphical abstract

A toddler developed severe contact dermatitis from her diabetes devices, leading to secondary infections and hospital admissions. This was followed by the development of a symmetrical exanthema with retroauricular and glutaeal distribution, interpreted as systemic contact dermatitis.

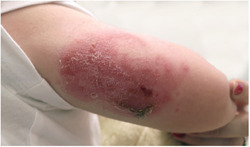

## 1 INTRODUCTION

Continuous glucose monitors (CGM) and insulin pumps have become the preferred treatment option for most young children and adolescents with type 1 diabetes (T1D), by avoiding fingerstick testing and providing real‐time glucose measurements. In Norway, the vast majority of T1D patients below the age of 18 are using both devices.^1^ Use of hapten‐containing diabetes devices and adhesives can, however, trigger allergic contact dermatitis.^2^ Colophonium, N,N‐dimethylacrylamide, ethyl cyanoacrylate, abitol and isobornyl acrylate (IBOA) are all documented in allergic contact dermatitis from diabetes devices, with IBOA being the most frequently reported.^3^, ^4^ Allergic contact dermatitis in patients dependent on glucose monitors and insulin pumps is an issue of growing concern, and IBOA was named ‘Allergen of the Year’ in 2020 by The American Contact Dermatitis Society.^2^, ^5^, ^6^ We describe a toddler who developed systemic contact dermatitis to IBOA and 4‐tert‐butylcatechol (PTBC) found in her diabetes devices.

## 2 CASE REPORT

An 8‐month‐old girl was diagnosed with T1D, and insulin treatment was initiated with insulin pump (Medtronic MiniMed 640G) in combination with CGM (Medtronic Guardian 3). The insulin pump application site was on the buttocks, alternating sides every 3 days. The application site of her CGM was the dorsal upper arm, alternating sides weekly. Four months after introducing the devices the patient developed dermatitis corresponding to the application sites of her CGM. Besides atopic eczema and an episode of impetigo, the patient was free of previous dermatoses and had no known allergies or hereditary dermatological disease.

**FIGURE 1:**
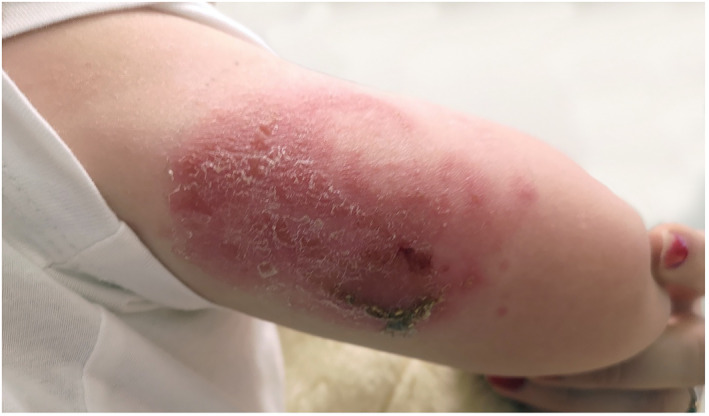
Allergic contact dermatitis located to application site of diabetes device (upper right arm). Photo: Ine Eriksen, University of Oslo.

The toddler was initially treated with topical hydrocortisone and antiseptics (fusidic acid and dibrompropamidine), and several adhesives were attempted, but without remission (Figure 1). The dermatitis remained unfluctuating, until 18 months later, when the patient developed a penicillin‐resistant *Staphylococcus aureus*‐infected dermatitis and lymphangitis on the dorsal side of her right upper arm corresponding to the application site of her glucose monitor. Topical regimens were intensified with fluocinolone acetonide with clioquinol. She was, nonetheless, hospitalised a month later due to fever (38.7°C) and further deterioration, as the infected dermatitis now affected both her chest, abdomen, and arms.

**FIGURE 2:**
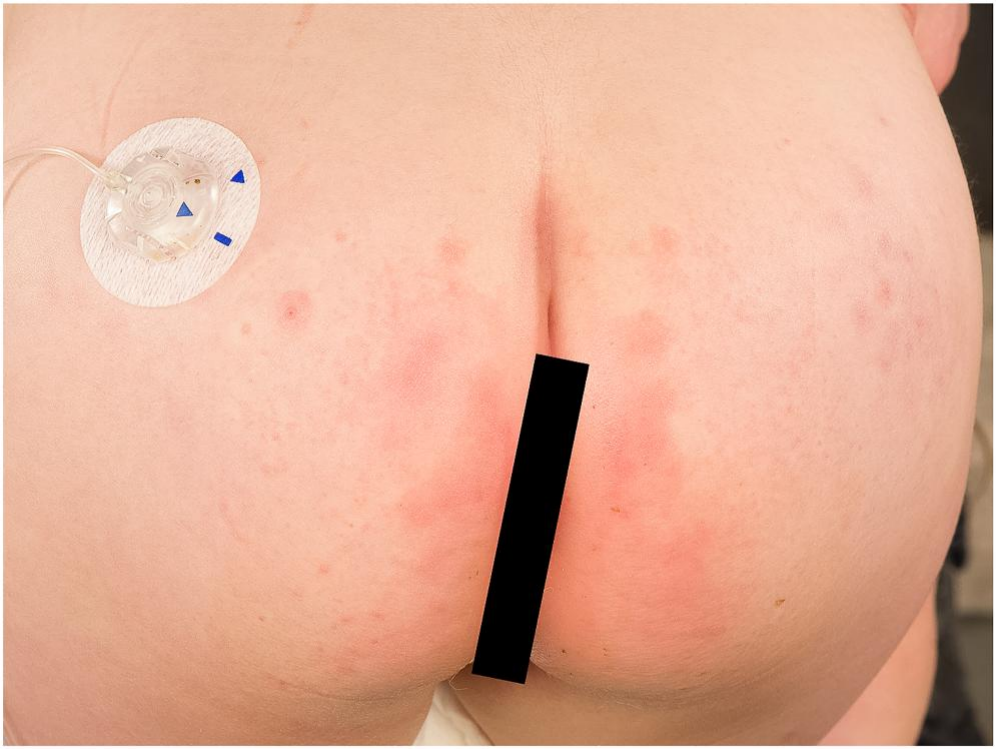
Systemic contact dermatitis presenting as symmetrical glutaeal exanthema, better known as ‘baboon syndrome’. Photo: Ine Eriksen, University of Oslo.

Upon admission, a symmetrical exanthema with retroauricular and glutaeal distribution was observed (Figure 2). Skin swabs from the upper arm were once again positive for penicillin‐resistant *Staphylococcus aureus*, while skin swabs from the diaper area were negative for both bacteria and yeast. Blood cultures were negative.

The patient was treated with intravenous cloxacillin, followed by oral administration of clindamycin. Topical fluocinolone acetonide with clioquinol prior to hydrocortisone butyrate were applied to the wide‐spread dermatitis. In the diaper area, monotherapy with clotrimazole was introduced. While the overall condition of the patient improved, the dermatitis located to her diabetes devices, as well as the symmetrical exanthema, persisted for a total of 3 months. Hence, systemic contact dermatitis was suspected, and the patient was referred to a dermatology department.

Patch testing was performed 7 weeks after hospitalisation with European Baseline Series (Modified S‐1000, without p‐Phenylenediamine and Epoxy resin/Bisphenol A), Plastics & Glues Series (PG‐1000, without Bisphenol A) and Isobornyl Acrylate 0.1% in pet. The test substances (Chemotechnique Diagnostics, Vellinge, Sweden) were applied to the upper back for 48 h in Finn Chambers (SmartPractice, Phoenix, Arizona) on Scanpor tape (Norgesplaster, Vennesla, Norway) and removed by the patient's parents. No test substances were diluted. The rash was still present at the time of patch testing.

Reading was done on day three (according to the guidelines of the European Society of Contact Dermatitis), obtaining positive and clinically relevant reactions (++) to both isobornyl acrylate and 4‐tert‐butylcatechol (PTBC).^7^

Based on the long‐lasting dermatitis to the application sites, we assumed that the toddler was exposed to IBOA and PTBC through the diabetes devices use. The presence of IBOA has previously been confirmed in the Medtronic devices and been reported to be responsible for allergic contact dermatitis.^8^ Consequently, Freestyle Libre 2 glucose sensor was attempted, with no skin reaction. The patient was however in need of a real‐time CGM with the possibilities of CGM/pump interaction. The providers of both Tandem T:slim X2 insulin pump (Rubin Medical) and Dexcom G6 CGM (Nordic Infucare) both confirmed on request that their products did not contain IBOA nor PTBC. These devices were introduced accordingly, and her long‐lasting dermatitis resolved at last. Of note, Dexcom G6 contains other identified allergens causing allergic contact dermatitis.^9^, ^10^

## 3 DISCUSSION

There is a wide array of terms/acronyms and clinical subtypes in describing systemic contact dermatitis. Baboon syndrome is perhaps the most well‐known and recognisable form; presenting with diffuse, well demarcated erythema of the buttocks, upper inner thighs, and axillae.^11^ SDRIFE (symmetrical drug‐related intertriginous and flexural exanthema) criteria includes exposure to a systemically administered drug (without previous cutaneous sensitisation), in addition to the absence of systemic symptoms.^11^ As the reported patient had not received any systemic drug, and was febrile upon admission, she was not a candidate for the SDRIFE diagnosis. We have chosen to describe this case with the general term ‘systemic contact dermatitis’, as the clinical presentation does not fully meet the diagnostic criteria of a specific subtype.

Systemic contact dermatitis is a condition that occurs when an individual sensitised to a contact allergen is exposed to the same allergen or a cross‐reacting molecule t hrough a systemic route.^12^ This elicits an immune response (commonly type IV delayed‐hypersensitivity) that produces cutaneous manifestations.^12^ Routes of systemic exposure to allergens include transcutaneous, transmucosal, oral, intravenous, intramuscular, and inhalational.^12^ We believe that our child was systemically exposed to the allergen(s) through the continuous use of diabetes devices on her atopic barrier‐defect skin.

The patch tests were positive for both IBOA and PTBC. The acrylic monomer IBOA is found in paints, ink, plastics, and rubber products, non‐pesticidal agricultural products like herbs and plants, as well as in adhesives and sealants.^5^, ^13^ IBOA causing allergic contact dermatitis in diabetes patients is now widely known.^2^ PTBC has, to our knowledge, not been described as a contributor to allergic contact dermatitis related to diabetes devices. This hapten can be found in products like rubber, paper, paints, adhesives, and oil derivates.^14^

Our diagnosis of systemic contact dermatitis was based on the history, positive patch tests, inadequate treatment responses, as well as the clinical presentation with symmetrical and intertriginous exanthemas located predominantly to the diaper area, but also retroauricular regions. None of these areas had been in contact with her diabetes devices, indicating a systemic response. Most important is that the dermatitis resolved with discontinuation of hapten‐containing diabetes devices.

We suspect that systemic contact dermatitis caused by CGMs and insulin pumps, as described in this case, is an underreported complication among patients with diabetes. Content information regarding device components and adhesives is not always readily available. Thus, reporting future cases is important in documenting the extent of this problem and accentuating the need for transparency and legislation changes.

## 4 CONCLUSION

The continuous hapten exposure through diabetes devices and their adhesives can lead to systemic contact dermatitis. We suspect that this is an underreported complication in diabetic patients. Manufacturers of advanced diabetes technology should be more transparent regarding content of device components and adhesives, to make it easier for patients and clinicians to choose tolerable products.

## CONFLICT OF INTEREST STATEMENT

The authors declare no conflicts of interest.

## AUTHOR CONTRIBUTIONS

**Anne Viktoria Lyngstadaas**: Writing – original draft (equal); Writing – review & editing (equal). **Jan‐Øivind Holm**: Writing – original draft (equal); Writing – review & editing (equal). **Lars Krogvold**: Resources (equal); Writing – original draft (equal); Writing – review & editing (equal). **Anne Karin Måløy**: Writing – review & editing (equal). **Christoffer Aam Ingvaldsen**: Investigation (equal); Project administration (equal); Writing – original draft; Writing – review & editing.

## ETHICS STATEMENT

Both parents have given their consent to the publication of this material.

## Data Availability

Data sharing is not applicable to this article as no new data were created or analyzed in this study.
